# A commentary on ‘Indications, trends, and perioperative outcomes of minimally invasive liver surgery in non-obese and obese patients: an international multicenter propensity score-matched retrospective cohort study of 9963 patients’

**DOI:** 10.1097/JS9.0000000000000399

**Published:** 2023-04-14

**Authors:** Xingru Wang

**Affiliations:** Department of Hepatobiliary Surgery, Qujing Second People’s Hospital of Yunnan Province, Qujing, People’s Republic of China

*Dear Editor*,

We read the study by Zimmitti *et al.* 1, published recently in the *International Journal of Surgery*, with great interest and pleasure. In this international multicenter retrospective cohort study of 9963 patients, the outcomes of nonobese and obese patients (BMI 18.5–29.9 and BMI ≥30, respectively) undergoing minimally invasive surgery (MILS) and open liver surgery (OLS) were compared using 1:1 propensity score matching (PSM) to minimize bias. The authors are praised for performing a well-designed international multicenter study to evaluate the safety and eventual benefits of MILS in obese patients. There are, however, some concerns regarding the study which I would like to discuss.

First, the authors classified the types of liver resection into three or more contiguous segments (e.g. trisegmentectomy, hemihepatectomy, and extended hepatectomy) based on the Brisbane 2000 Terminology for Liver Anatomy and Resections. However, this does not quantify the complexity of liver resection. The difficulty of MILS depends not only on the technical complexity of liver resection but also on various factors such as the patient’s liver background, tumor size and location, or the degree of liver fibrosis^2^. It is well known that complex hepatolithiasis, large tumors or tumors located close to major large vessels of the liver may affect the surgeon’s decision, as minimally invasive liver resection requires more technical training and experience in MILS. We noted a significantly higher proportion of complex procedures; longer operative time; higher incidence of overall, liver-related, and serious postoperative complications and mortality; and higher positive margin rates in OLS patients than in MILS patients in this study. Is it possible that the data were biased because the aforementioned factors influenced the choice of surgeon between MILS and OLS? In addition, it is worth exploring whether MILS has oncological benefits equivalent to those of OLS in patients with liver malignancies. Oncological benefits are the ultimate measures of whether a technology is beneficial. Unfortunately, we did not observe relevant oncologic data or the short-term and long-term outcomes of the patients in this study. We believe that the supplementation of survival analysis of obese and nonobese patients with malignant liver tumors in MILS and OLS, liver function-related indicators such as reserve function assessment, and relevant quantitative indicators of liver resection complexity such as the IWATE (Iwate Watanabe-type classification of liver resection difficulty) classification would make the findings more persuasive.

Despite these problems, this study may be the first large international multicenter case cohort study to examine the benefits of minimally invasive liver surgery in obese and nonobese patients. We thank the authors for their significant contributions.

## Ethical approval

Not applicable.

## Patient consent

Not applicable.

## Sources of funding

This study did not receive any funding.

## Author contribution

X.W.: conceptualization and writing – original draft and critical revision.

## Conflicts of interest disclosure

The author completed the ICMJE uniform disclosure form. The author declared no conflicts of interest for this article.

## Research registration unique identifying number

Not applicable.

## Guarantor

Xingru Wang.
